# The P286R mutation of DNA polymerase ε activates cancer-cell-intrinsic immunity and suppresses endometrial tumorigenesis via the cGAS-STING pathway

**DOI:** 10.1038/s41419-023-06418-3

**Published:** 2024-01-18

**Authors:** Ming Tang, Shasha Yin, Hongliang Zeng, Ao Huang, Yujia Huang, Zhiyi Hu, Ab Rauf Shah, Shuyong Zhang, Haisen Li, Guofang Chen

**Affiliations:** 1 Shanghai Key Laboratory of Maternal Fetal Medicine, Shanghai Institute of Maternal-Fetal Medicine and Gynecologic Oncology, Clinical and Translational Research Center, Shanghai First Maternity and Infant Hospital, School of Medicine, Tongji University, Shanghai, 200092 China; 2 Department of Biochemistry and Molecular Biology, Hollings Cancer Center, Medical University of South Carolina, Charleston, SC USA; 3 Center of Medical Laboratory Animal, Hunan Academy of Chinese Medicine, Changsha, 410013 China; 4 Department of Pathology and Microbiology, UNMC, Omaha, USA; 5 Key Laboratory of Prevention and Treatment of Cardiovascular and Cerebrovascular Diseases, Gannan Medical University, Ministry of Education, Ganzhou, 341000 China; 6 School of Basic Medicine, Gannan Medical University, Ganzhou, 341000 China; 7 School of Life Sciences, Fudan University, Shanghai, 200438 China; 8 AoBio Medical Co., Shanghai, 200438 China

**Keywords:** Mechanisms of disease, Cancer

## Abstract

Endometrial carcinoma (EC) is a prevalent gynecological tumor in women, and its treatment and prevention are significant global health concerns. The mutations in DNA polymerase ε (POLE) are recognized as key features of EC and may confer survival benefits in endometrial cancer patients undergoing anti-PD-1/PD-L1 therapy. However, the anti-tumor mechanism of POLE mutations remains largely elusive. This study demonstrates that the hot POLE P286R mutation impedes endometrial tumorigenesis by inducing DNA breakage and activating the cGAS-STING signaling pathway. The POLE mutations were found to inhibit the proliferation and stemness of primary human EC cells. Mechanistically, the POLE mutants enhance DNA damage and suppress its repair through the interaction with DNA repair proteins, leading to genomic instability and the upregulation of cytoplasmic DNA. Additionally, the POLE P286R mutant also increases cGAS level, promotes TBK1 phosphorylation, and stimulates inflammatory gene expression and anti-tumor immune response. Furthermore, the POLE P286R mutation inhibits tumor growth and facilitates the infiltration of cytotoxic T cells in human endometrial cancers. These findings uncover a novel mechanism of POLE mutations in antagonizing tumorigenesis and provide a promising direction for effective cancer therapy.

## Introduction

The endometrial carcinoma (EC) arises from abnormal endometrial epithelium and is one of the most prevalent malignant cancers in the female reproductive system. Its incidence and mortality have been steadily increasing in the past years [1, 2]. Currently, the EC accounts for approximately 50% of newly diagnosed gynecological malignancies [2]. However, traditional chemotherapy and immunotherapy have limitations in treating endometrial cancer patients. Notably, immune checkpoint inhibitors, such as PD-1 antibody, are reported to be ineffective among 60–80% of cancer patients [3]. The ECs have been classified into four distinct subgroups based on their molecular characteristics: POLE mutation, microsatellite instability, copy number low, and copy number high [4, 5]. The POLE protein, which contains both polymerase and exonuclease activities, plays a crucial role in DNA replication and repair, thereby ensuring low mutation rates in dividing cells [6, 7]. Genetic mutations leading to functional POLE deficiency result in the accumulation of mutant genes and the initiation of tumorigenesis. Mutations within the POLE exonuclease region have been associated with improved survival in endometrial cancer patients receiving immune checkpoint inhibitor treatment [8–10] and have been identified as valuable prognostic factors [11, 12]. However, the mechanisms by which POLE exonuclease mutations counteract tumorigenesis and enhance PD-1 inhibitor sensitivity remain unclear.

The inability of POLE mutants to maintain genomic replication fidelity dramatically increases the mutation rate, leading to unpredictable genome modifications such as DNA deletions and insertions [13, 14]. Genomic DNA damage during mitosis stress leads to the release of nuclear DNA into the cytosol, serving as a significant source of cytosolic nucleic acids. The accumulation of cytosolic DNA results in the activation of the cyclic GMP-AMP synthase (cGAS). The cGAS quits from its inactive status and activates the stimulator of interferon genes (STING) by generating the second messenger cyclic GMP-AMP [15–17]. The activated STING further stimulates the TBK1-IRF3 signaling cascade to induce inflammatory gene expressions. Thus, the cGAS-STING signaling pathway functions as the core player of the cellular immune system [18, 19], and its activation represents a promising direction for the development of anti-tumor medicines. The activation of the cGAS-STING pathway has been shown to enhance immune responses and inhibit carcinogenesis in various tumor models [20–23], suggesting its anti-tumor potential. Nevertheless, the regulation of the cGAS-STING signaling pathway by POLE mutations remains poorly understood.

Our study demonstrates that POLE mutations result in genome instability and reduced DNA damage repair ability in human EC cells. We also observed the activation of the cGAS-STING pathway by POLE mutation in vitro and in vivo. Furthermore, the POLE mutation suppressed the tumor growth of human EC cells in humanized immune mice by enhancing the infiltration and activation of cytotoxic T cells. Our findings provide valuable insights into the anti-tumor effects of POLE mutations and offer potential strategies for the effective treatment of human ECs.

## Results

### POLE P286R mutation activates innate immune signaling pathway in human EC

**Fig. 1:**
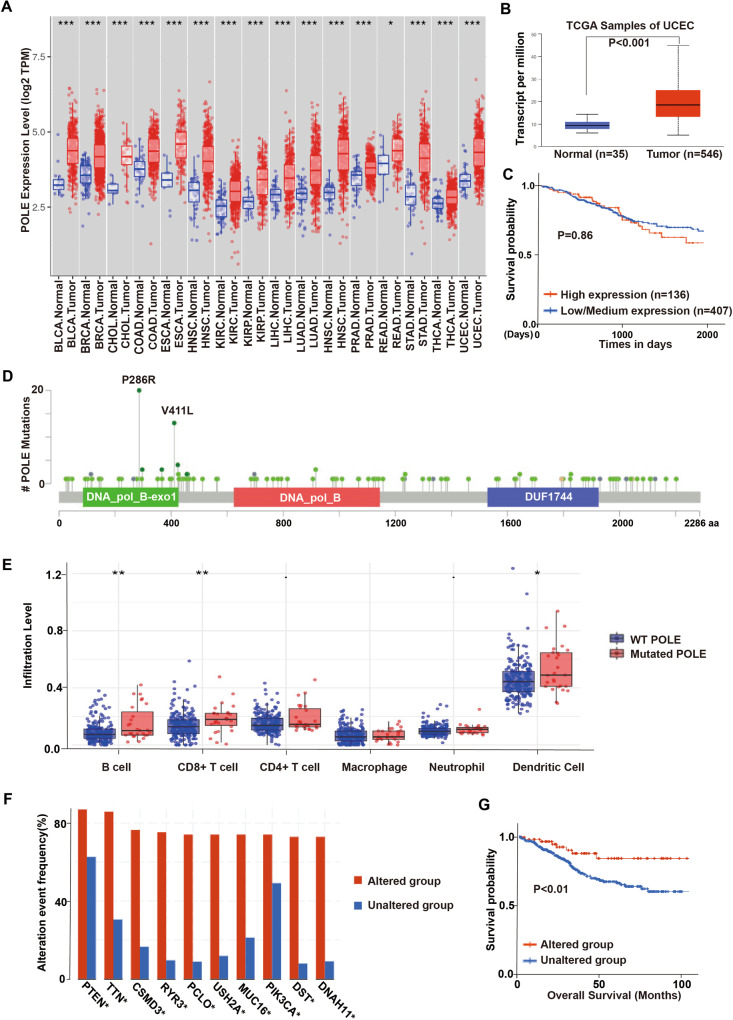
POLE mutations elicit immune activation in endometrial cancer. **A** The expression levels of POLE in cancers and their adjacent normal tissues from the TCGA database of TIMER website (BLCA bladder urothelial carcinoma, BRCA breast invasive carcinoma, CHOL cholangiocarcinoma, COAD colon adenocarcinoma, ESCA esophageal, HNSC head and neck squamous cell carcinoma, KIRC kidney renal clear cell carcinoma, KIRP kidney renal papillary cell carcinoma, LIHC liver hepatocellular carcinoma, LUAD lung adenocarcinoma, HNSC Head and Neck squamous cell carcinoma, PRAD prostate adenocarcinoma, READ rectum adenocarcinoma, STAD stomach adenocarcinoma, THCA thyroid carcinoma, UCEC uterine corpus endometrial carcinoma). **B** The mRNA level of POLE in normal tissues and ECs from the TCGA database. **C** Kaplan–Meier survival curves of endometrial cancer patients with high or low POLE expression level. Both high and low POLE groups were defined by the median expression value of the POLE transcript among the study population. The EC patients whose POLE level exceeded the median value were termed high expression, whereas the other patients with low POLE expression levels were named low expression. **D** Schematics of the POLE proteins show the positions of individual somatic mutations identified in the endometrial cancer TCGA cohort. **E** The correlation between *POLE* mutations and the infiltration abundance of immune cells (B cell, CD4^+^ T cell, CD8^+^ T cell, macrophage, neutrophil, and dendritic cell) in endometrial cancer. **F** The comparison of immune infiltration level among tumors with somatic copy number alterations by the SCNA module. **G** Kaplan–Meier survival curves of EC patients with *POLE* mutations or WT *POLE*. Data are shown as mean ± SD. *p < 0.05, **p < 0.005, ***p < 0.001 significantly different from Control.

To investigate the pathological significance of POLE mutations in endometrial carcinogenesis, we analyzed the POLE expression profile in various endometrial cancer datasets via the cBioPortal Cancer Genomics database. The expression levels of the POLE in tumors were found to be significantly higher than in normal adjacent tissues (Fig. 1A). Consistently, endometrial cancers showed elevated POLE transcript levels in comparison to normal tissues (Fig. 1B). However, a high POLE expression in endometrial cancer patients did not correspond to improved survival compared to those with low POLE expression (Fig. 1C). Additionally, the POLE expression level did not show any correlation with the clinicopathologic phenotypes such as tumor grade and clinical stage in endometrial cancer patients (Data not shown). We collected 596 endometrial carcinoma specimens from patients visiting our hospital, among which approximately 13.14% were found to harbor POLE mutations. Through bioinformatics analysis of sequencing data, we identified 157 distinct POLE mutations from the 596 endometrial carcinoma specimens (Supplementary Table 1). These POLE mutations were dispersed throughout the *POLE* gene (Fig. 1D). The most common mutation observed was the P286R mutation in exon 9 of the human *POLE* gene, found in 20 patient samples, while another hotspot mutation, V411L, identified in 13 patient cases. Notably, we observed a positive correlation between POLE mutations and the infiltration abundance of various immune cells, including T cell, B cell, and Dendritic cells in endometrial cancer (Fig. 1E). The somatic copy number alteration (SCNA) module further demonstrated a positive association between immune infiltration levels and POLE mutations in endometrial cancer (Fig. 1F). Importantly, the patients with POLE alterations showed favorable survival when compared to those with wild-type (WT) POLE (Fig. 1G). Collectively, these findings suggest that POLE has a high mutation frequency in endometrial cancer, and its mutations positively correlate with immune infiltration levels.

### POLE mutations suppress cancer stemness and trigger intrinsic immunity in primary EC cells

**Fig. 2:**
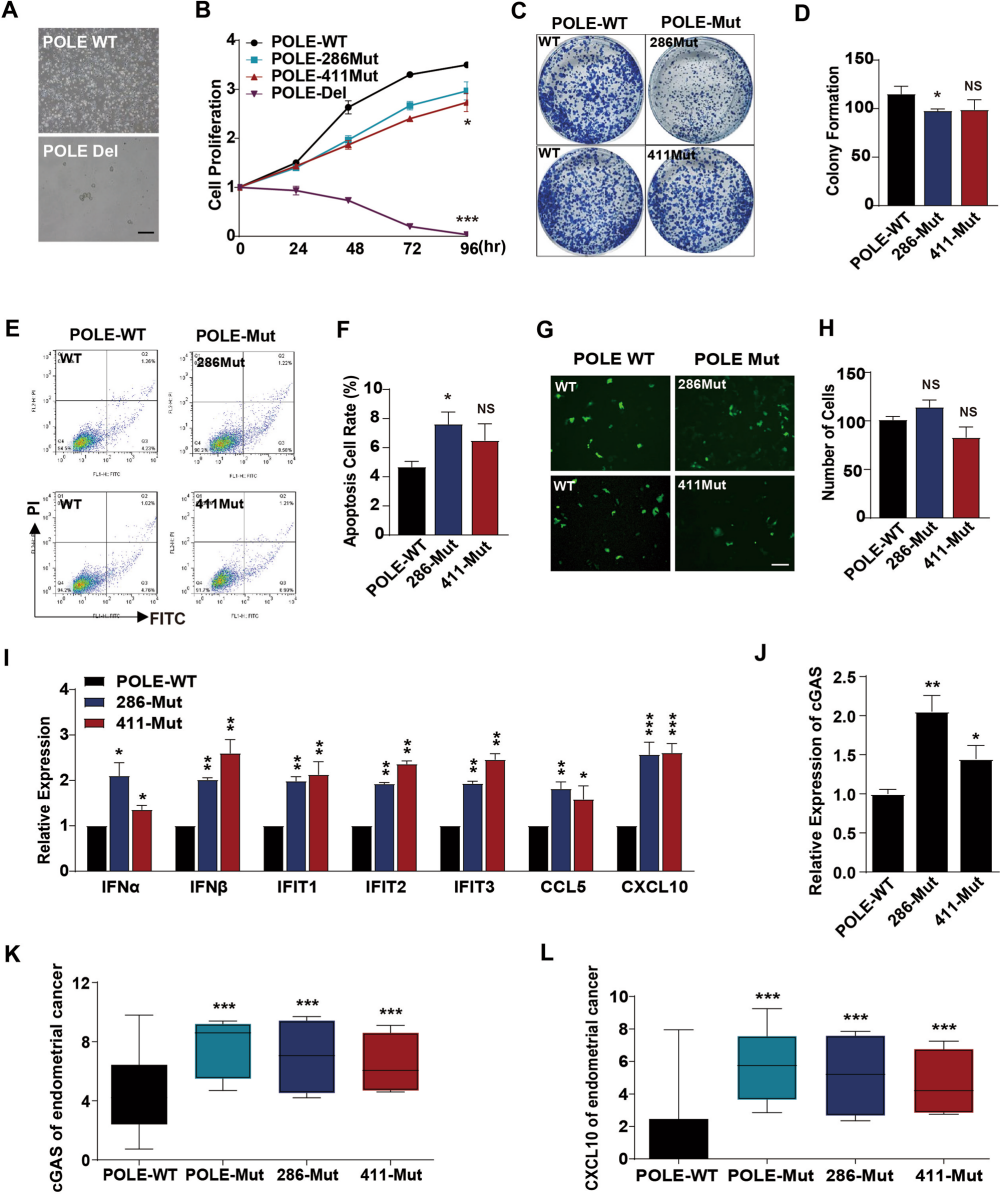
POLE mutations impede the proliferation and activate inflammatory responses in human EC cells. **A** Representative morphologies of WT EC cells and the engineered EC cells with *POLE* deletion. **B** CCK8 analyses of WT EC cells and the engineered EC cells with *POLE* deletion or mutation. **C** Colony formation assay of WT EC cells and the engineered EC cells with POLE mutation. **D** Quantification of colony number of EC cells treated as (**C**). **E** Apoptosis analysis of EC cells with POLE mutant or WT POLE. **F** Quantification of the percentages of apoptotic cells in the (**E**). **G** Representative images of transwell migration analysis of WT EC cells and the engineered EC cells with POLE deletion or mutation. **H** Quantification of migrated cells in the (**G**). **I** Relative mRNA levels of inflammatory cytokines (*IFN*, *IFIT*, *CCL5*, and *CXCL10*) in WT EC cells and POLE mutant cells. **J** RT-PCR analysis of *cGAS* gene in WT EC cells and POLE mutant cells. **K** Relative mRNA level of *cGAS* gene in human EC specimens with POLE mutations or WT POLE. **L** Relative expression level of *CXCL10* gene in human ECs with POLE mutation or WT POLE. Data are shown as mean ± SD. *p < 0.05, **p < 0.005, ***p < 0.001 significantly different from Control.

To investigate the influences of POLE mutations on EC growth, we endeavored to establish stable EC cell lines carrying the P286R or V411L mutation using SpCas9-driven homology-directed repair system (Supplementary Fig. S1A–C). Genome sequencing analysis confirmed the precise insertion of the P286R mutation in exon 9 and the V411L mutation in exon 13 (Supplementary Fig. S1D, E). Additionally, we attempted to generate stable EC cells with POLE deletion. However, POLE deletion resulted in the death of EC cells (Fig. 2A), preventing the establishment of stable POLE knockout cells. Both P286R and V411L mutations were found to slightly inhibit the proliferation of EC cells when compared to those with WT POLE (Fig. 2B). Furthermore, these POLE mutations were observed to suppress the colony formation ability of EC cells (Fig. 2C, D), indicating a reduction in the stemness of EC cells with POLE mutation. Subsequent experiments demonstrated that these mutations promoted the apoptosis of EC cells (Fig. 2E, F), potentially accounting for their decreased proliferation and stemness. Notably, the transwell migration assay revealed that POLE mutations had no significant effect on cell migration (Fig. 2G, H). Notably, EC cells harboring the POLE P286R or V411L mutation exhibited higher expression levels of inflammatory cytokines and chemokines *IFN*, *IFIT*, *CCL5*, and *CXCL10* (Fig. 2I), as well as the *cGAS* gene (Fig. 2J). Consistently, the expression levels of the *cGAS* and *CXCL10* genes were higher in human EC samples with POLE mutations when compared to those with WT POLE (Fig. 2K, L). These findings indicate that POLE mutations promote the expression of inflammatory genes and the core component of the cGAS signaling pathway.

### POLE mutations induce genomic DNA damage in primary EC cells

**Fig. 3:**
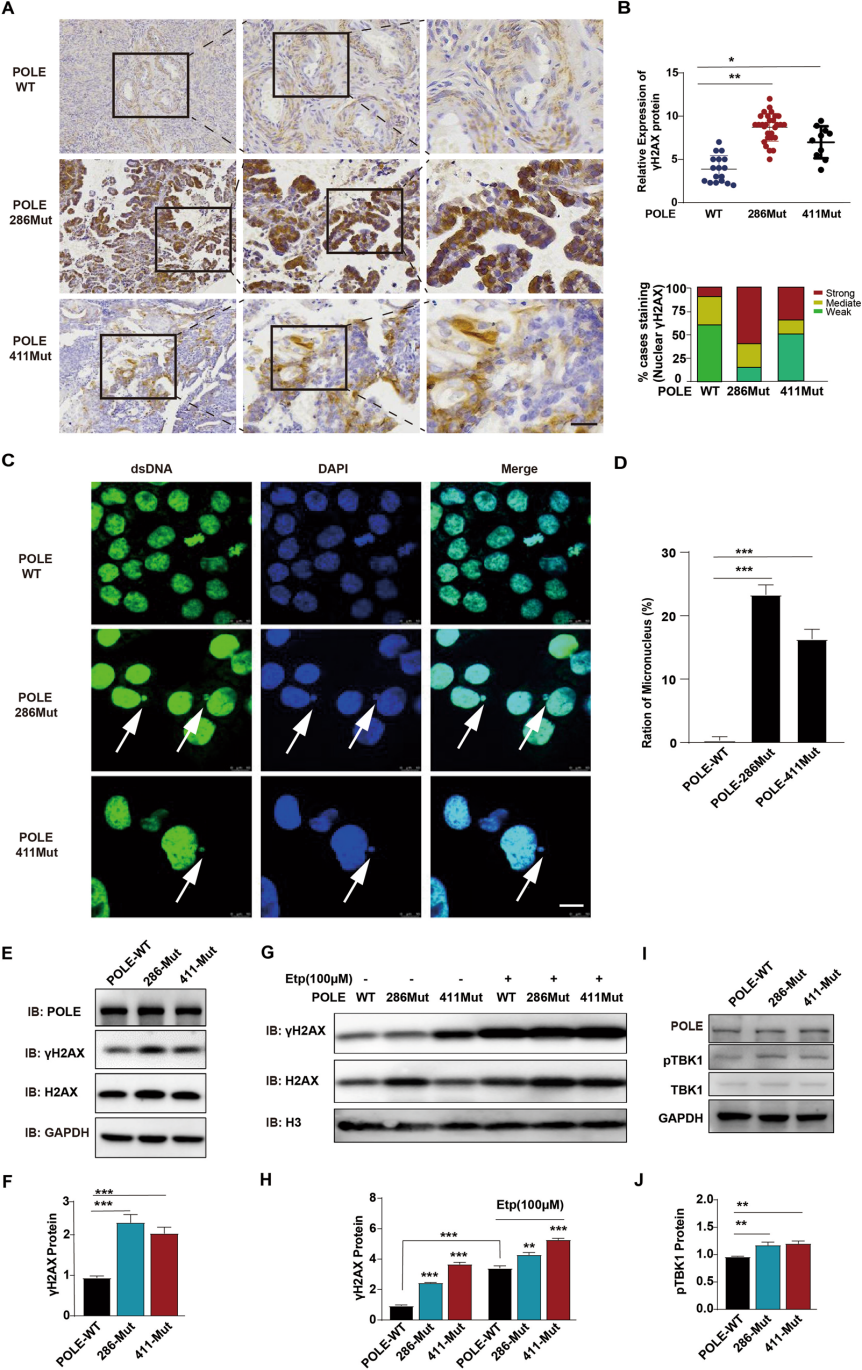
POLE mutations promote the occurrence of endogenous DNA breaks. **A** Immunohistochemical analysis of γH2AX expression in human EC specimens. The images are sequentially magnified from left to right and the black frames indicate the amplification area. Scale bar, 100 μm. **B** Quantification of nuclear γH2AX staining intensity in the (**A**). **C** Immunofluorescence analysis of micronucleus in human EC cells with WT POLE or P286R mutation. Scale bar, 25 μm. **D** Quantification of genomic damage by the micronucleus assay. **E** Western blot analyses of γH2AX and H2AX proteins in human EC cells with POLE mutation or WT POLE. **F** Quantification of γH2AX and H2AX expression levels in the EC cells treated as in (**E**). **G** Western blot analyses of γH2AX and H2AX proteins in human EC cells bearing POLE mutant or WT POLE in the absence or presence of Etoposide (100 μM). **H** Quantification of γH2AX expression level in the EC cells treated as in (**G**). **I** Western blotting of TBK1 and its phosphorylation in human EC cells with WT or mutant POLE. **J** Quantification of TBK1 phosphorylation level in the EC cells treated as in (**I**). Data are shown as mean ± SD. *p < 0.05, **p < 0.005, ***p < 0.001 significantly different from Control.

To investigate the impact of POLE mutations on DNA damage, we conducted an analysis of DNA double-strand breaks (DSBs) markers in human EC samples and primary EC cells with or without POLE mutations. Compared to EC samples with WT POLE, those with the POLE P286R mutation exhibited significantly higher expression levels of the classical DSB marker γH2AX (Fig. 3A, B). Furthermore, extranuclear DNA aggregates were observed in EC cells with POLE P286R mutation, indicating the accumulation of damaged chromatin following the POLE mutation (Fig. 3C, D). Western blotting analysis revealed that both P286R and V411L mutations led to a significant increase in the total γH2AX protein level (Fig. 3E, F). Considering the inhibitory effect of the well-known anticancer drug etoposide on topoisomerase II activity, we sought to determine whether the combination of etoposide and POLE mutation could induce increased DNA damage in human EC cells. Consistent with its ability to enhance DNA damage in human breast and adrenal cancer cells [24, 25], etoposide was found to elevate the levels of the DSB marker γH2AX in primary EC cells (Fig. 3G, H). Notably, the promotion of γH2AX levels by POLE mutations was further enhanced by the addition of chemotherapeutic agent etoposide, suggesting that the combination of POLE mutation and etoposide might exhibit more potent anticancer effects. Given that TBK1 phosphorylation is a crucial event in the cGAS-STING signaling pathway, we investigated the influence of POLE mutations on TBK1 activation. Consistent with their promotion of cGAS expression, POLE mutations were found to increase TBK1 phosphorylation levels in ECs (Fig. 3I, J), indicating the activation of the cGAS-STING pathway after POLE mutation. These findings demonstrate that POLE mutations induce DNA damage in primary EC cells.

### POLE P286R mutation decreases the DSB repair ability and increases cytosolic DNA accumulation

**Fig. 4:**
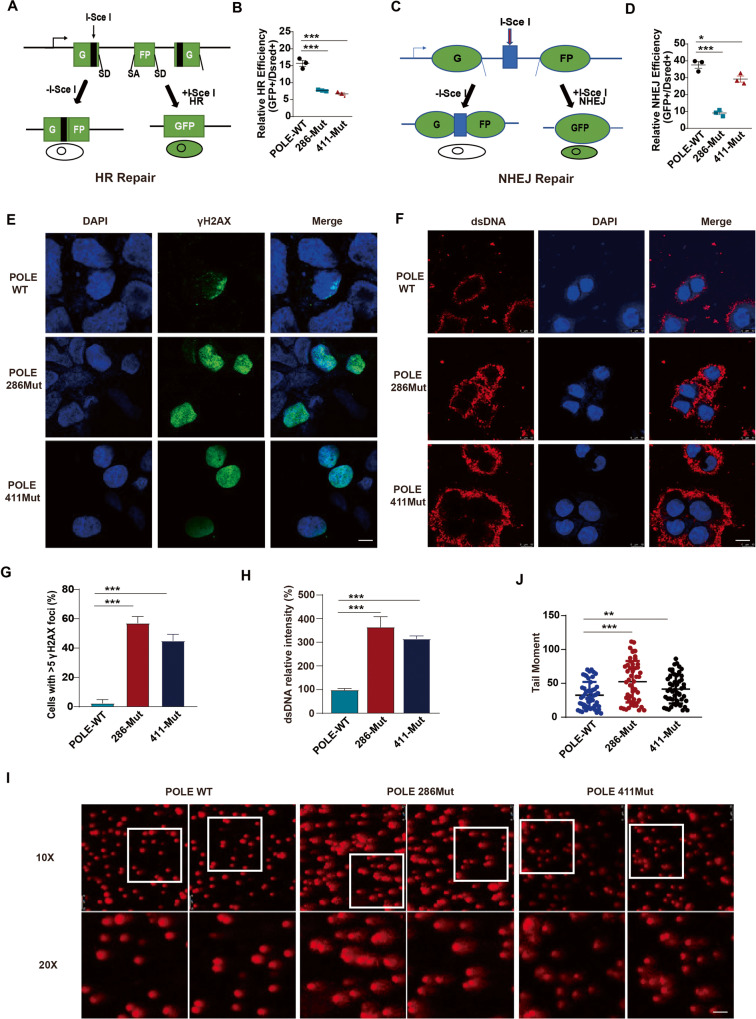
POLE P286R mutation suppresses the DSB repair and increases cytosolic DNA. **A** Schematic for the fluorescent reporter assay to determine the homologous recombination (HR) pathway. The correction of the DSBs in the target sequence allows GFP expression. **B** Quantification of the efficiency of HR repair pathway in cells transfected with WT or mutant POLE. **C** Illustration of fluorescent reporter assay to measure the non-homologous end joining (NHEJ) pathway. **D** Quantification of the efficiency of NHEJ repair system in cells treated with WT or mutant POLE. **E** Immunofluorescence analysis of γH2AX level in human EC cells with POLE mutation or WT POLE. Scale bar, 25 μm. **F** Immunofluorescence staining of dsDNA level in human EC cells with POLE mutation or WT POLE. Scale bar, 25 μm. **G** Quantification of nuclear γH2AX expression level in the EC cells treated as in (**E**). **H** Quantification of dsDNA intensity in the EC cells treated as in (**F**). **I** Comet assay of human EC cells with POLE mutations or WT POLE. **J** Quantification of tail moments in the comet assay. Data are shown as mean ± SD. *p < 0.05, **p < 0.005, ***p < 0.001 significantly different from Control.

Given the critical role of POLE exonuclease in maintaining DNA replication fidelity, we then sought to investigate how POLE mutations change genomic damage repair. Utilizing a sensitive fluorescent reporter assay, we measured the efficiency of homologous recombination (HR) or non-homologous end joining (NHEJ) by introducing the rare-cutting I-SceI endonuclease-induced DNA double-strand breaks (DSBs) with 3′ cohesive ends at the target locus [26, 27]. Successful DSB repair through the HR or NHEJ pathway leads to the reconstitution and expression of the GFP reporter; our findings indicated that both P286R and V411L mutants notably decreased the percentage of GFP-positive cells compared to the WT POLE (Fig. 4A–D), indicating a reduction in the repair efficiency of both HR and NHEJ pathways by POLE mutations. Consequently, the level of DSB marker γH2AX in EC cells was increased by the POLE P286R and V411L mutations (Fig. 4E, G). Additionally, the amount of double-strand DNA (dsDNA) in the cytoplasm was also enhanced by POLE P286R and V411L mutations (Fig. 4F, H), suggesting the leakage of intranuclear DNA into the EC cytosol. Furthermore, single-cell gel electrophoresis comet assay revealed that POLE P286R and V411L mutations resulted in increased genomic DNA damage in individual EC cells (Fig. 4I, J). These findings collectively indicate that POLE mutations impair DSB repair capacity and lead to an increase in cytosol DNA levels.

### POLE P286R mutation promotes inflammatory chemokine expressions by regulating DNA damage and repairing signaling

**Fig. 5:**
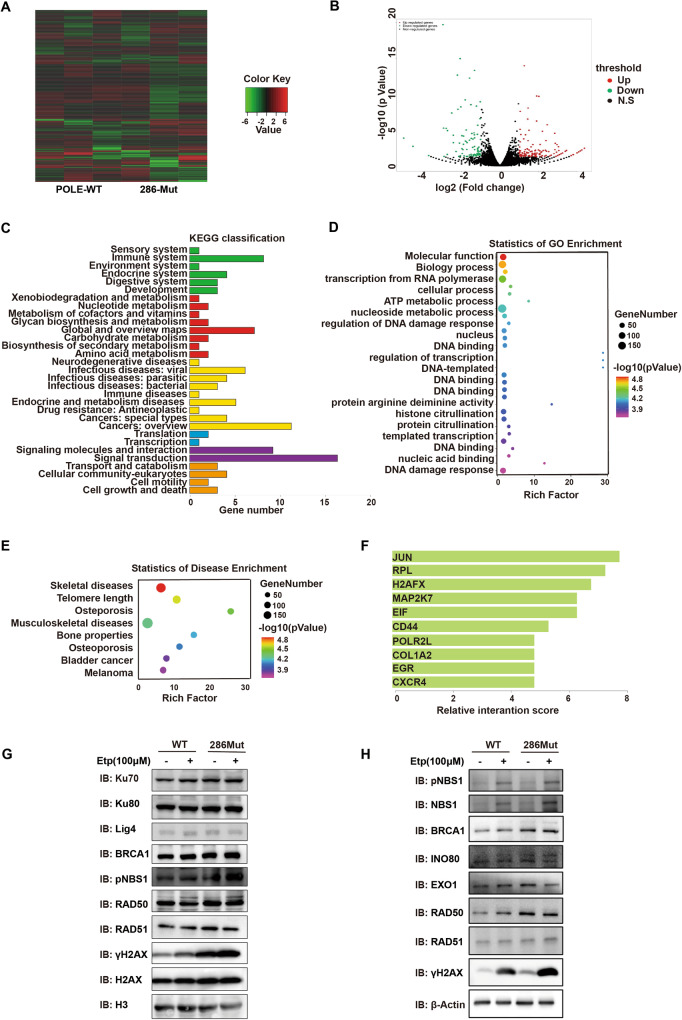
POLE P286R mutation regulates the DSB repair pathway. **A** Heatmap analyses of gene expression profiles in human EC specimens with WT or mutant POLE. **B** GSEA of the EMT gene set in the EC cells with P286R mutation or WT POLE. The hallmark EMT genes were obtained from the Molecular Signatures Database. **C** KEGG pathway analysis of the EMT gene set in EC cells with P286R mutation or WT POLE. **D** GO analyses of the differentially expressed genes in human EC samples with POLE mutations or WT POLE. **E** GO analyses of the differentially expressed genes in human EC samples with POLE mutations or WT POLE. **F** Relative scores of the predicated interaction proteins in human EC cells with POLE mutations. **G** Western blot analyses of repair-related proteins in human EC cells and mutant POLE cells without or with Etoposide treatment. **H** Western blot analyses of repair-related proteins in human EC cells and mutant POLE cells without or with Etoposide treatment.

To gain deeper insights into the impact of POLE mutation in human EC cells, we conducted transcriptome sequencing in EC cells with WT POLE or the P286R mutation. Since POLE P286R mutation could induce stronger effects in the induction of genomic DNA breaks, cGAS expression, and EC apoptosis compared to the POLE V411L mutation (Figs. 2E, F, J, K, 3C–E, and 4C–E), we next focused on studying the downstream molecular mechanisms of the P286R mutant. Bioinformatic analysis revealed that POLE mutations altered the expression profiles of numerous functional genes (Fig. 5A), among which some genes were involved in the DNA binding and genomic damage regulation being upregulated by the mutation (Fig. 5B). Subsequent KEGG pathway, gene ontology (GO) and disease enrichment analyses further supported that a large number of functional genes were enriched in categories related to DNA damage, DNA binding, transcription, and related disease (Fig. 5C–E). Furthermore, protein interaction analysis in POLE mutation EC cells revealed a high interaction potential with DNA damage pathway proteins, particularly the DNA damage marker H2AFX (Fig. 5F). Evaluation of the regulation of POLE mutations on important molecules participating in HR or NHEJ pathway indicated that POLE mutations selectively increased the expressions of repair-related protein RAD50, Ku70, and Ku80 in EC cells (Fig. 5G, H), while the levels of other factors such as EXO1 and NBS1 were not affected by POLE mutations (Fig. 5H). The etoposide treatment had no influence on repair-related and most other proteins except the NBS1 (Fig. 5G, H). Interestingly, Co-immunoprecipitation (co-IP) experiments demonstrated a direct binding between POLE and RAD50 (Supplementary Fig. S2A), with their interaction remaining unchanged by the etoposide treatment (Supplementary Fig. S2A). Additionally, the interaction between PARP and POLE was weakened by etoposide (Supplementary Fig. S2A), and p-STING and p-TBK1 were detected in the Co-IP product of the POLE protein (Supplementary Fig. S2B), suggesting potential interaction. Taken together, these findings suggest that POLE mutation reduces the capacity of EC cells to repair genomic damage by interacting with DNA damage repair protein RAD50.

### POLE P286R mutation promotes antitumor immunity and inhibits tumor growth by activating cGAS-STING signaling

**Fig. 6:**
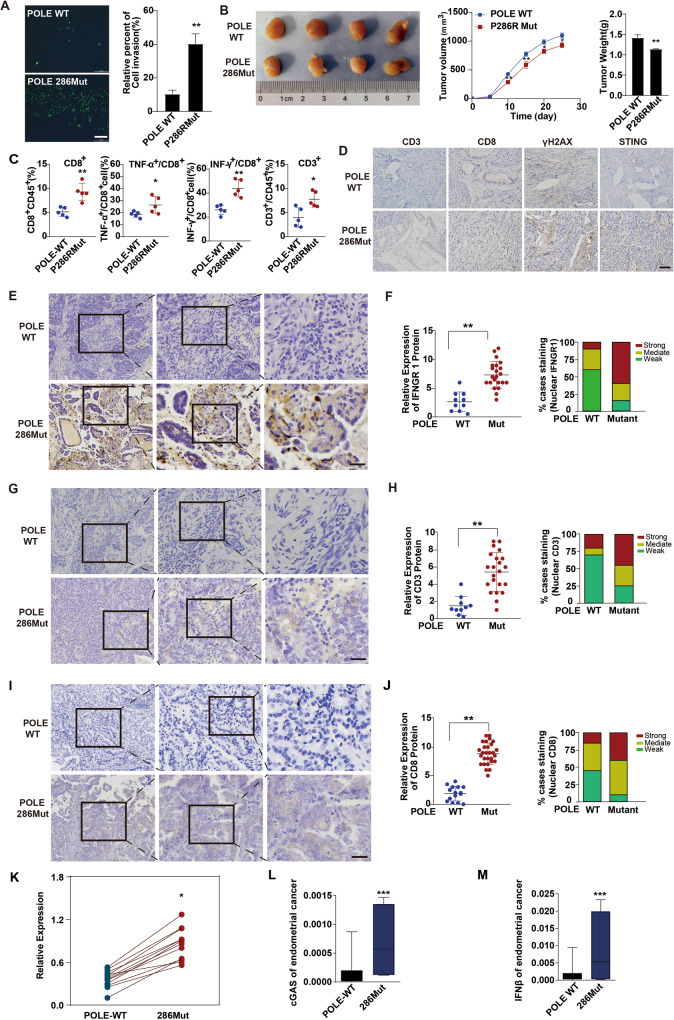
POLE P286R mutation enhances immune cell infiltration and activates the cGAS-STING pathway in human endometrial carcinomas. **A** Representative images of transwell migration assay of T cells cocultured with WT EC cells or mutant POLE cells. **B** Tumorigenicity assessment of human EC cells with POLE P286R mutation or WT POLE in the humanized huHSC-NCG mice. Left: representative tumor pictures; Middle: tumor growth curves; Right: tumor weights. **C** Flow cytometry analysis of the percent of parent immune cells in the above tumors with POLE P286R mutation or WT POLE. **D** Representative images of IHC staining of CD3, CD8, γH2AX, and STING proteins in above tumors with POLE P286R mutation or WT POLE. Scale bar, 50 μm. **E** Immunohistochemical analysis of IFNGR1 expression in human EC specimens. The images are sequentially magnified from left to right, and the black frames indicate the amplification area. Scale bar, 100 μm. **F** Quantification of IFNGR1 expression intensity in (**E**). **G** Immunohistochemical staining of CD3 expression in human EC specimens. The pictures are sequentially magnified from left to right, and the black frames suggest the amplification area. Scale bar, 100 μm. **H** Quantification of CD3 staining intensity in (**G**). **I** Immunohistochemical analysis of CD8 expression in human EC specimens. The images are sequentially magnified from left to right, and the black frames indicate the amplification area. Scale bar, 100 μm. **J** Quantification of the intensity of CD8 staining in (**I**). **K** Quantification of STING expression level in human EC specimens with POLE P286R mutation or WT POLE. **L** Quantification of cGAS level in human EC specimens with WT or mutant POLE. **M** Quantification of IFNβ expression level in human EC specimens with WT or mutant POLE. Data are shown as mean ± SD. *p < 0.05, **p < 0.005, ***p < 0.001 significantly different from Control.

**Fig. 7:**
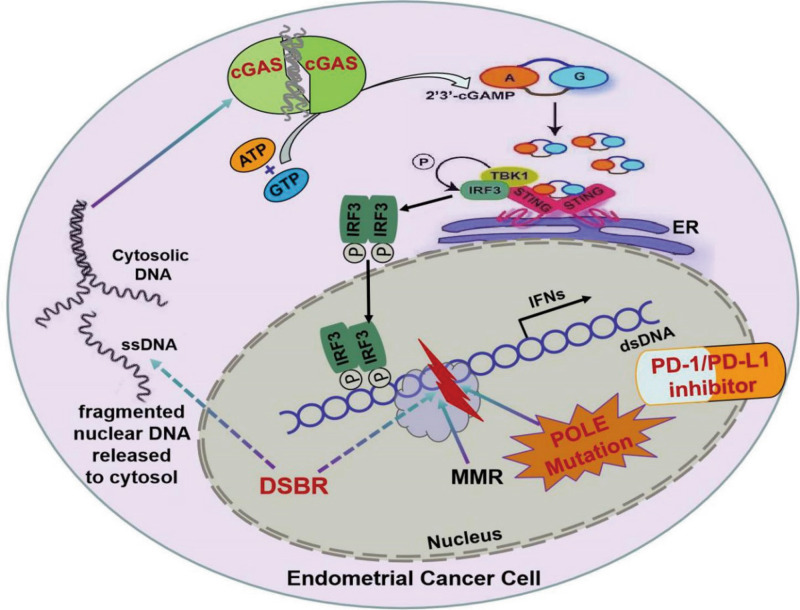
Graphical abstract. POLE mutations regulate DNA double-strand breaks repair and trigger an immune response and antitumor activity through STING/TBK1/IRF3 signaling pathway.

Given the critical role of infiltrating and activated cytotoxic T cells in antitumor immunity [28, 29], we next sought to investigate the impact of POLE mutation on tumor growth and T cell infiltration. Using a co-culture transwell system, we observed that EC cells with POLE P286R mutation attracted more migrated T cells in comparison to WT EC cells, suggesting an enhancement of the mutation in tumor cell migration of T cells (Fig. 6A). Additionally, the in vivo tumor growth analysis in huHSC-NCG humanized mice revealed that the POLE P286R mutation suppressed tumor growth, leading to reduced tumor size and weight (Fig. 6B). Flow cytometry analysis of these tumors indicated increased numbers of CD8^+^ and CD3^+^ T cells in tumors with the POLE P286R mutation when compared to WT POLE tumors (Fig. 6C). Moreover, the percentages of IFN-γ^+^ CD8^+^ and TNF-α^+^ CD8^+^ T cells were significantly elevated in tumors with the POLE P286R mutation, indicating the promotion of cytotoxic T cell infiltration and function (Fig. 6C). Immunohistochemistry staining further confirmed increased infiltration of tumor-infiltrating CD8^+^ and CD3^+^ T lymphocytes in tumors with POLE P286R mutation (Fig. 6D). Consistent with our previous observations (Figs. 2J, K and 3A–F), the tumors with POLE P286R mutation exhibited higher levels of γH2AX and STING proteins, (Fig. 6D), suggesting increased DNA damage and cGAS-STING pathway activation. Furthermore, immunohistochemical analyses revealed significantly higher levels of IFN-γ receptor in POLE P286R mutant samples compared to WT POLE tumors (Fig. 6E, F). Human EC samples with the POLE mutation showed higher expression levels of CD3 and CD8 proteins when compared to control specimens, suggesting enhanced invasion of functional T lymphocytes in human EC tumors (Fig. 6G–J). Upon the enhancement of POLE mutation on genomic damage and TBK1 phosphorylation in vitro and in humanized mouse xenograft models, we measured the expressions of core components of the cGAS-STING pathway in human EC samples with or without the POLE P286R mutation. The expression levels of cGAS, STING, and the inflammatory cytokine IFNβ were notably upregulated in POLE P286R mutation EC specimens, indicating the activation of downstream immune signaling of the GAS-STING pathway (Fig. 6K–M). In conclusion, these results suggest that POLE mutations may impede tumor growth and trigger anticancer immune responses through the STING/TBK1/IRF3 signaling pathway (Fig. 7).

## Discussion

The traditional classification of the ECs based on histopathology is limited in distinguishing molecular characteristics and predicting of therapeutic outcomes. ECs exhibit significant heterogeneity in terms of genotype, morphology, and clinical stage. Recent advancements in next-generation sequencing technologies, such as whole exosome sequencing, have identified a wide array of genetic mutations in EC tumorigenesis [30, 31]. Among these genetic variations, POLE exonuclease mutations have been identified in approximately 7–15% of all EC cases [32, 33], establishing POLE mutations as a defining feature of a primary EC subtype. It is important to note that POLE mutation is closely associated with a favorable prognosis for endometrial cancer patients [12]. However, the underlying molecular mechanisms remain largely unclear. Our findings demonstrate that POLE mutations lead to increased genomic damage, enhanced cGAS level, upregulated TBK1 phosphorylation, and promoted inflammatory gene expressions in human EC cells. The activation of the cGAS-STING pathway by POLE mutation likely contributes to the advantageous survival of endometrial cancer patients with POLE mutations, as the cGAS-STING pathway has been associated with anti-tumor activity through the stimulation of inflammatory genes [20, 21]. Given the involvement of POLE mutants in most tumors [34], further exploration is warranted to determine whether POLE mutations also activate the cGAS-STING pathway in other cancer models. Nonetheless, the selective activation of the cGAS-STING pathway and the stimulation of cancer-cell-intrinsic immunity by POLE mutations provide a theoretical-foundations for accurate molecular typing and personalized therapy of malignant tumors.

The majority of somatic POLE mutations are missense substitutions, accounting for over 80% of all mutant cases [35]. These point mutations are randomly distributed across the *POLE* gene and include the known hotspot P286R and V411L mutants. Given the crucial role of POLE exonuclease in DNA proofreading and repair, its disruption by the P286R and V411L mutations leads to the accumulation of DNA replication errors and defective mismatch repair, resulting in ultra-mutation. Our study confirms that both POLE P286R and V411L mutations significantly reduce the activity of HR- or NHEJ-driven repair pathways in primary human EC cells. Notably, the P286R mutation exerts a more pronounced inhibitory effect on the NHEJ repair process compared to the V411 mutant. Beyond the high mutational burden caused by these mutations, this intrinsic attenuation of DNA damage repair in tumor cells with POLE mutation exacerbates genomic replication stress and chromosomal instability. The accumulation of damaged DNA in the nucleus, as evidenced by the substantial cytosolic dsDNA in EC cells with the POLE P286R mutant, could trigger the cGAS and initiate its downstream signaling and inflammatory responses. It is noteworthy that several DNA damage repair factors, including RAD50 and PARP1, appear to interact with the POLE protein. The P286R mutation’s interference with DNA binding, possibly altering the secondary structure of the POLE molecule [36, 37], suggests a stronger binding with repair proteins, potentially detaining them at POLE-binding sites rather than at the DNA damage locus. However, further research is needed to explore this speculation in pathological conditions and understand how POLE mutants activate cGAS signaling. Additionally, our study revealed that POLE mutations significantly enhanced cGAS expression level in primary human cells and tumor specimens. Considering POLE’s essential role in heterochromatin maintenance [38, 39], it is plausible that the POLE mutant promotes the transition from heterochromatin to euchromatin around the *cGAS* gene locus. However, further investigations are necessary to confirm the occurrence of this phenomenon and elucidate occurs and its molecular mechanisms.

The POLE mutation triggers an intrinsic inflammation response in primary human EC cells, leading to the activation of cGAS signaling and rapid TBK1-mediated phosphorylation of the transcription factor IRF3, subsequently resulting in the transcription of interferons and other inflammatory genes. In this study, we observed a significant upregulation of inflammatory IFN and IFIT family genes in primary EC cells with POLE mutations, which may play a crucial role in suppressing EC proliferation and expansion. Furthermore, the levels of chemotactic cytokines, such as CCL5 and CXCL10, were markedly higher in the EC cells with POLE mutations. These chemokines are known to induce the migration and activation of various immune cells, including macrophages and T cells, in the extracellular space [40–42]. Our findings strongly suggest that CCL5 and CXCL10 from EC cells with POLE mutations facilitate the migration and infiltration of cytotoxic lymphocytes by activating their receptors on immune cells. This is supported by the observation of higher amounts of CD3^+^ and CD8^+^ T cells in EC specimens with POLE P286R mutant compared to WT POLE samples. These data align with previous observations and provide key insights that may explain the improved survival of endometrial cancer patients with POLE mutations. However, the specific tumor-derived cytokine responsible for the recruitment of cytotoxic T cells into endometrial cancers remains unclear. Therefore, further studies are needed to elucidate how POLE mutations regulate chemokine secretion from tumor cells and promote the invasion of immune cells in solid tumors. The enhanced genomic damage and immune activation associated with POLE mutations suggest that cancers with POLE mutation may be more sensitive to immune checkpoint blockade therapy. This potential sensitivity underscores the promise of small molecule inhibitors selectively targeting POLE exonuclease activity, which could significantly enhance the therapeutic efficiency of anti-PD-1/PD-L1 treatment in carcinomas with WT POLE. In summary, our findings highlight the potential of POLE mutation as a predictive biomarker for immune checkpoint blockade therapy and suggest targeted inhibition of POLE exonuclease activity as a promising approach to enhance the efficacy of anti-PD-1/PD-L1 treatment in cancers with WT POLE. These insights may have significant implications for the development of personalized treatment strategies for endometrial cancers and other solid tumors.

## Materials and methods

### Specimen collection from cancer patients

The objectives and procedures of this study were viewed and approved by the Scientific and Ethical Committee of the Shanghai First Maternity and Infant Hospital, School of Medicine, Tongji University. All the experiments were in line with the ethical standards of the 1964 Declaration of Helsinki and its later amendments or comparable ethical standards. The patients with endometrial cancer visited the Department of Gynecology in our hospital, and their tumor grade and stage were classified by at least two pathologists following the International Union Against Cancer guidelines. The clinicopathological features of cancer patients are summarized in Supplementary Table 1. The patients with pregnancy, immunosuppressive therapy, chemotherapy, radiotherapy, or other antitumor treatments were excluded from the study. The written informed consent was obtained from each participant and her family. Five hundred ninety-six tumor specimens were isolated from the patients at the time of surgical biopsy.

### Identification of POLE gene mutations

A portion of tumor samples was cut into small pieces and digested at 56 °C overnight in tissue lysis buffer. Genomic DNA was then isolated using a DNA extraction Kit and amplified with specific PCR primers. The target products were about 300 bp and spanned the mutation sites. Sanger sequencing was then performed to measure the POLE mutation. The number, size, and frequency of mutations within the *POLE* gene in all 596 cases were analyzed by sequence comparison.

### Immunohistochemical analysis

A portion of the tumors was embedded in the paraffin and then cut into 10-μm sections using a microtome. The sections were transferred onto the slides and fixed in 4% paraformaldehyde for 20 min. Following the permeation, the sections were washed three times and stained with primary antibodies at 4 °C overnight. After three washes, the sections were successively incubated with secondary antibodies and DAPI for 3 h. The coverslips were then applied with permanent synthetic mounting media. All pictures were observed and taken under an inverted microscope.

### RNA-seq and gene expression analysis

Total RNAs were extracted from tumor specimens using the TRIzol reagent (Invitrogen). After the enrichment with poly-A selection, mRNAs were prepared for RNA-seq libraries based on the manufacturer’s protocol. RNA-seq library was then deeply sequenced using Illumina HiSeq2000, and the sequencing data were analyzed.

### Plasmid construction and lentivirus production

The pU6-sgRNA-EF1α-SpCas9-Puro-WPRE and POLE-sgRNA-SpCas9-Knockout vectors were purchased from Hanyin Biotechnology Limited Company (Shanghai). The sgRNAs targeting POLE gene mutations were designed using the CCTOP tool (https://cctop.cos.uni-heidelberg.de:8043/). The lentivirus particles expressing SpCas9 and sgRNAs were provided by the Hanyin Biotechnology Limited Company.

### Establishment of endometrial carcinoma cells with POLE mutation

The endometrial cancer cells were infected with lentivirus encoding both SpCas9 and sgRNAs at a proper ratio. Two days later, the fluorescence-positive cells were sorted out by BD FACSAria™ III Sorter and then seeded on a 100-mm culture dish. After a week, a single cell colony was picked and transferred to the 12-well culture plate. After being subjected to genome sequencing, the desired cells were further expanded.

### Cell culture

The Ishikawa, RL95-2, and Hec1B cells were cultured in DMEM/F12 medium supplemented with 10% fetal bovine serum (FBS) and 1% penicillin/ streptomycin, while the ECC1 cells grew in RPMI-1640 medium containing 10% FBS and 1% penicillin/streptomycin. All above cells were maintained at 37 °C, 5% CO_2_.

### RT-PCR

Total mRNAs from cancer cells were reversely transcribed into cDNAs with a High-Capacity RNA-to-cDNA Kit (#R223-01, Vazyme). The PCR reaction was performed with the One-Step SYBR® PrimeScript®PLUS RT-RNA PCR Kit. The relative expression levels of target genes were normalized to the internal gene *Gapdh*.

### Western blotting

Samples were dissociated in RIPA lysis buffer containing a proteinase inhibitor cocktail. Once protein concentration was determined with the BCA Protein Assay Kit, total proteins were separated on SDS-PAGE gels and transferred onto the PVDF membranes. After blocking, the membranes were incubated overnight with primary antibodies at 4 °C. Primary antibodies were listed below: γH2AX (#2577S, Cell Signaling Technology), H2AX (#7631, Cell Signaling Technology), H3 (#17168-1-AP, Proteintech), POLE (#132100, Genetex), pTBK1 (#5483, Cell Signaling Technology), TBK1 (#BM4038, Boster), cGAS (#15102, Cell Signaling Technology), Ku70 (#A0883, ABclonal), Ku80 (#A5862, ABclonal), Lig4 (#A1743, ABclonal), RAD50 (#A3078, ABclonal), RAD51 (#A2829, ABclonal), BRCA1 (#A11034, ABclonal), NBS1 (#7703, ABclonal), p95/NBS1 (#ab23996, Abcam) EXO1 (#A1941, ABclonal), PARP1 (#A2432, ABclonal), ATR (#A7247, ABclonal), p-STING (#50907T, Cell Signaling Technology), STING (#ab92605, Abcam), β-Actin (#AC026, Abcam), and GAPDH (#ab9485, Abcam). Following three washes, the membranes were probed with HRP-conjugated secondary antibodies at room temperature (RT) for 1 h. The target proteins were viewed with an ECL Plus Kit (Abcam).

### Chromatin fractionation

The cells were digested into a single-cell solution and then centrifuged at 10,000 rpm for 30 s. The pellet was resuspended in 500 μL buffer I containing protease inhibitor cocktail and later centrifuged at 13,000 rpm for 3 min. After two-time washes in buffer I, the pellet was mixed with 1× SDS loading buffer and subsequently boiled for 10 min.

### Co-immunoprecipitation assay

Single-cell solutions were centrifuged at 10,000 rpm for 30 s. The cell pellets were resuspended in 1 mL lysis buffer on ice for 30 min and immediately sonicated using a microtip at 35% amplitude 10 times. These samples were centrifuged at 12,000 rpm for 15 min, and their supernatants were collected for the addition of specific antibodies and protein A/G agarose beads. After the incubation at 4 °C overnight with a rotary shaker, the immunoprecipitants were washed three times and boiled in 1× SDS loading buffer for 5 min. Following the centrifugation, the supernatants were subjected to western blotting.

### Immunofluorescence staining

The cells were fixed in 4% paraformaldehyde for 15 min and then permeabilized with methanol at −20 °C for 10 min. After the blocking, cells were stained with primary antibodies at 4 °C overnight. Following three-time washes, the cells were probed with TRITC/FITC-conjugated secondary antibodies for 1 h at RT. After DAPI staining, the images were observed under a fluorescence microscope (Leica, Germany).

### Comet assay

The cells were mixed with low-temperature-melting agarose solution at a ratio of 1:1 and then spread on glass slides. The slides were put in pre-cooled lysis buffer at 4 °C for 2 h and subsequently electrophoresed in another running buffer for 20 min. After the propidiumiodide staining, the slides were observed and pictured under a fluorescence microscope.

### Colony formation assay

The equal cells were plated in each well of a 6-well dish and then cultured at 37 °C, 5% CO_2_ for 2 weeks. These cells were stained with crystal violet and then captured under the Olympus CK30-F200 microscope.

### Engrafted tumor model

All experimental procedures on the mice had been approved by the Committee of Animal Care and Utilization of Tongji University. The huHSC-NCG (CH) mice were obtained from GemPharmatechTM and housed in a constant temperature (24–26 °C) and humidity (40–60%) room with a 12-h light-dark cycle. Human EC cells were resuspended in sterile PBS and subcutaneously injected into the flank of 4-week-old NSG mice at the dosage of 1 × 10^6^ cells/mice. The length and width of tumors were detected every 5 days by a manual caliper, and the tumor volume was calculated using the formula: ½ × longitudinal diameter (length) × the greatest transverse diameter (width)^2^. When the tumor volume reached 2000 mm^3^, mice were euthanized, and the tumors were harvested for further analyses.

### Flow cytometry analysis

Tumors were dissociated into single cells and were then filtrated through 70 μm cell strainers. Single cells were resuspended in cell staining buffer (#FXP005, 4abio) and then stained with fluorochrome-conjugated antibody combinations at appropriate concentration. The antibody information are shown as follows: PerCP-Cy5.5-Anti-CD45 (#103131, Biolegend), PE-Cy7-Anti-CD4 (#100421, Biolegend), FITC-Anti-CD8 (#ab237367, Abcam), APC-Anti-TNF-α (#506307, Biolegend), PE-Anti-IFN-γ (#505807, Biolegend), PerCP-Cy5.5-Anti-CD45R (#ab210342, Abcam), and PE-Anti-CD3 (#ab22268, Abcam). DAPI (#D9542, Sigma) was added to exclude dead cells. After washing, the stained cells were analyzed on a BD Fortessa machine. The data were processed with FlowJo software.

### Transwell migration assay

The co-culture of human EC cells and T cells was performed in the 24-well culture plate with 0.4 μm-pore polyester membrane insert (#3412, Corning). In brief, tumor cells with POLE WT or P286R mutant were seeded into the bottom of a 24-well plate at a concentration of 1 × 10^5^ cells/well and then cultured for 24 h. The next day, the transwell chamber was inserted into each well of the above 24-well plate, and T cells were then added to the upper chamber at the concentration of 5 × 10^4^ cells. After one day, the transwell chambers were collected and stained with the crystal violet. The migration of T cells was observed and imaged with an Olympus microscope.

### Statistical analysis

Quantification was calculated from at least three independent experiments and shown as mean ± SD. The statistical significance was determined by the equivalent non-parametric tests or by one-way or two-way analysis of variance (ANOVA). P-value (<0.05) was thought to be significant (not significant, *p* > 0.05; *p < 0.05, **p < 0.01, ***p < 0.001, and ****p < 0.0001). GraphPad was used to analyze all data.

### Supplementary information

Supplementary figures and tables

## Data Availability

Data are available on reasonable request. The data used to support the findings of this study are available from the corresponding author on request.
