# Supplementary material for: The P286R mutation of DNA polymerase ε activates cancer-cell-intrinsic immunity and suppresses endometrial tumorigenesis via the cGAS-STING pathway
Source: Cell Death Dis. 2024 Jan 18;15(1):69. doi: 10.1038/s41419-023-06418-3 (PMC10796917; doi:10.1038/s41419-023-06418-3)
Supplement: Supplementary file 1 — Supplementary figures and tables [file 41419_2023_6418_MOESM1_ESM.docx]

**Supplementary figures and tables**


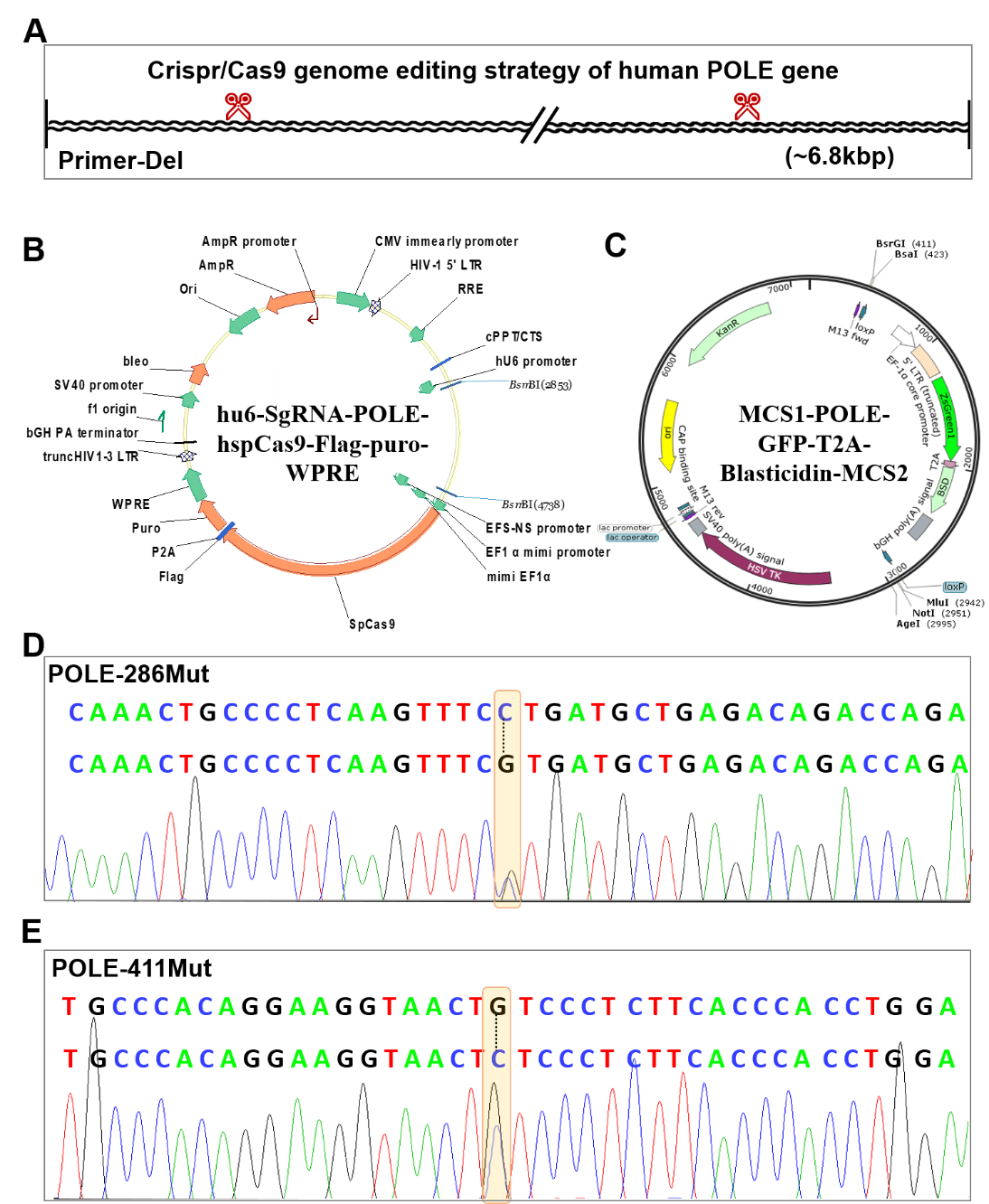


**Supplementary Figure S1. Establishment of human EC line with POLE mutation.**

**(A)** Schematic for the binding position of hEx30 sgRNA-4 Construction of human POLE gene editing Crisper/Cas9 plasmid strategy. **(B)** Illustration of the pU6-sgRNA-EF1α-SpCas9-Puro-WPRE vector with POLE mutation. **(C)** Illustration of the sgRNA-ZsGreen plasmid containing sgRNA that targets POLE P286R or V411L mutation. **(D)** Representative sequencing chromatogram of the P286R mutation in human *POLE* gene. **(E)** Genomic sequencing chromatogram of the POLE V411L mutation.

**
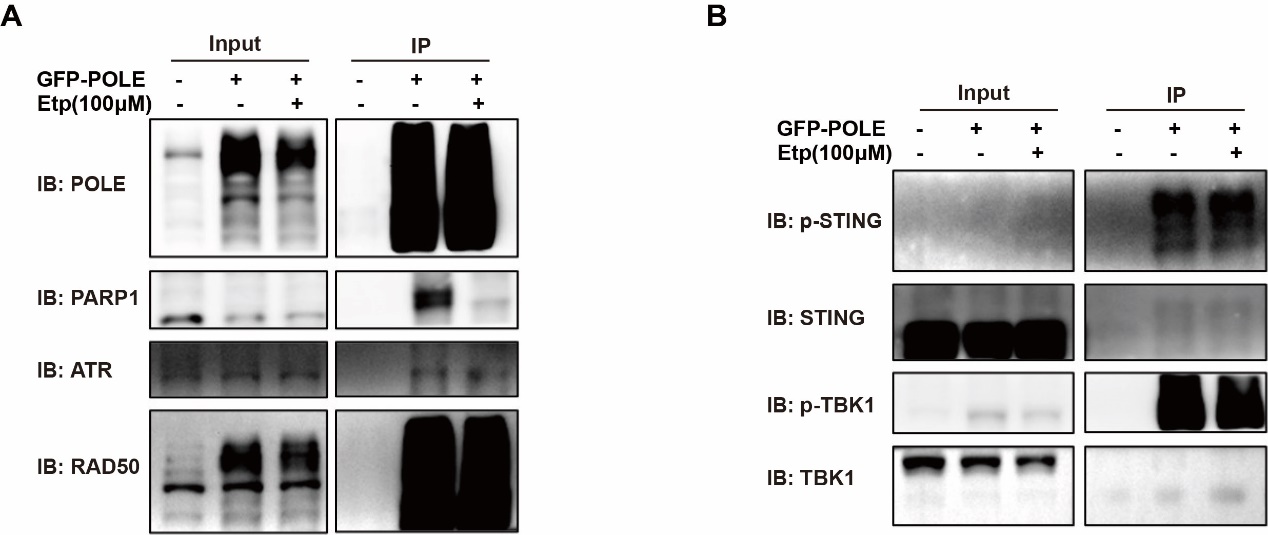
Supplementary Figure S2. POLE regulate the DSB repair pathway.**

**(A)** Western blot analyses of POLE, PARP1, ATR, and RAD50 proteins in whole cell lysates or anti-GFP immunoprecipitates of HEK293FT cells with GFP-POLE transfection or/and Etoposide treatment. **(B)** Western blot analyses of STING and TBK1 proteins in whole cell lysates or anti-GFP immunoprecipitates of HEK293FT cells with GFP-POLE transfection or/and Etoposide treatment.

**Table S1. The POLE mutations between the curettage samples and whole uterus sample of endometrial cancer with clinical features (n = 472).**

| Characteristic | | **Number of samples** | | **Number of mutations** | **Mutation Rate** | | | **Mutation Site** | **Mutation Frequency** |
| --- | --- | --- | --- | --- | --- | --- | --- | --- | --- |
| Total | 472 | | | 62 | 13.14% | | |  |  |
| Curettage Samples | | 262 | | 33 | | 12.60% | **P286R**  **V411L** | | **9**  **4** |
|  | | |  |  | |  | V474I | | 3 |
|  | | |  |  | |  | K472R | | 2 |
|  | | |  |  | |  | A428S | | 1 |
|  | | |  |  | |  | A456P | | 1 |
|  | | |  |  | |  | D443G | | 1 |
|  | | |  |  | |  | T483S | | 1 |
|  | | |  |  | |  | K425E | | 1 |
|  | | |  |  | |  | L283P | | 1 |
|  | | |  |  | |  | M447V | | 1 |
|  | | |  |  | |  | P441L | | 1 |
|  | | |  |  | |  | T279A | | 1 |
|  | | |  |  | |  | T457S | | 1 |
|  | | |  |  | |  | V464I | | 1 |
|  | | |  |  | |  | T279I | | 1 |
| Whole uterus Sample | | | 210 | 29 | | 13.81 % | **P286R**  **V411L** | | **3**  **3** |
|  | | |  |  | |  | T457M | | 2 |
|  | | |  |  | |  | Q303R | | 2 |
|  | | |  |  | |  | P441L | | 2 |
|  | | |  |  | |  | S297F | | 1 |
|  | | |  |  | |  | N423K | | 1 |
|  | | |  |  | |  | F285L | | 1 |
|  | | |  |  | |  | T279A | | 1 |
|  | | |  |  | |  | D301N | | 1 |
|  | | |  |  | |  | T457L | | 1 |
|  | | |  |  | |  | T278S | | 1 |
|  | | |  |  | |  | P286S | | 1 |
|  | | |  |  | |  | F285S | | 1 |
|  | | |  |  | |  | K429R | | 1 |
|  | | |  |  | |  | A456T | | 1 |
|  | | |  |  | |  | V411M | | 1 |
|  | | |  |  | |  | P452S | | 1 |
|  | | |  |  | |  | T278P | | 1 |
|  | | |  |  | |  | P476L | | 1 |
|  | | |  |  | |  | T449M | | 1 |
|  | | |  |  | |  | L 424 V | | 1 |
